# Assessment of non-communicable disease risk factors, functional performance, and health-related quality of life in adults: a comparative analysis in low-resourced urban and rural areas of South Africa

**DOI:** 10.1186/s12889-024-18964-2

**Published:** 2024-06-12

**Authors:** Fhatuwani W Ramalivhana, Tamrin Veldsman, Sarah J Moss

**Affiliations:** 1 Physical Activity, Sport, and Recreation Research Focus Area (PhASRec), Faculty of Health Sciences, North-West University, Potchefstroom, South Africa; 2 School of Health and Medical Sciences, University of Southern Queensland, Ipswich, Australia; 3 North-West University, Private Bag X6001, Potchefstroom, 2531 South Africa

**Keywords:** Functional performance, Health-related quality of life, Non-communicable diseases, Rural, Low-resourced urban, Urban

## Abstract

**Background:**

Globally, disparities between non-communicable disease (NCD) risk factors, functional performance, and health-related quality of life (HRQoL) exist in people living in rural and low-resourced urban settings. Evidence of these health differences determined with objective NCD risk factors and functional performance measurements in South Africa, is scarce. Therefore, the study aimed to determine the differences in NCD risk factors, functional performance and HRQoL between rural and low-resourced urban areas.

**Methods:**

The study recruited 311 adults (35–80 years) presenting with at least one NCD risk factor from low-resourced urban- (*n* = 183) and rural (*n* = 128) communities. Objective measurements of physical activity (PA) by means of combined heart rate and accelerometery, body composition employing skinfolds, peripheral lipid and glucose concentrations, blood pressure, functional performance indicators (handgrip, single leg stand, sit-to-stand, timed-up-and-go speed, predicted peak VO_2_ max); and HRQoL were measured according to standard procedures. Independent t-tests, Mann-Whitney U, and chi-square tests were performed to determine differences between the variables of low-resourced urban and rural settings.

**Results:**

The participants from the low-resourced urban setting were significantly older than the rural residents (59.1 ± 10.7 years vs. 52.8 ± 11.3 years; *p* = 0.001). NCD risk factors were significantly more prevalent in the low-resourced urban participants compared to rural participants, in particular for elevated systolic (85.8% vs. 62.5%; *p* = 0.001), and diastolic blood pressure (88.5% vs. 65.6%; *p* = 0.001), physical inactivity (95.9% vs. 87.7%; *p* = 0.026), increased cholesterol concentrations (22.1% vs. 8.7%; *p* = 0.002), and increased waist circumference (61.9% vs. 49.2%; *p* = 0.027). Low-resourced urban residents presented with a higher average body fat percentage (27.69% ± 7.65% vs. 12.23% ± 4.67%; *p* < 0.001), and lower moderate to vigorous PA levels (37.19 ± 49.55 [95% CI = 29.12–45.27] vs. 62.92 ± 60.43 min/week [95% CI = 47.95–77.90]; *p* = 0.003) compared to rural residents. Rural residents showed significantly better functional performance, including peak VO_2_ (23.99 ± 9.89 vs. 16.95 ± 7.64 ml/min/kg; *p* = 0.001) and single leg stand (right leg: 44.96 ± 18.47 vs. 20.87 ± 19.18 s; *p* = 0.001) as well as higher HRQoL for the physical (51.06 ± 8.14% vs. 45.62 ± 11.13%; *p* < 0.001) and mental (54.75 ± 8.24% vs. 48.91 ± 12.27%; *p* < 0.001) component scores compared to participants from the low-resourced urban areas.

**Conclusion:**

NCD risk factors, functional performance, and HRQoL significantly differ in rural communities compared to low-resourced urban communities in South Africa. Urban areas’ most prevalent risk factors were elevated blood pressure, physical inactivity, and increased waist circumference. Participants from rural areas demonstrated significantly better functional performance, such as fitness and balance. HRQoL was better in rural settings than in urban settings. Future intervention programmes should be tailored for specific settings.

## Background

Non-communicable diseases (NCDs) are a major global public health concern, leading to premature mortality, decreased functional capacity, disability, poor quality of life (QoL) and increased healthcare costs [1]. Each year, of all global deaths, 74% (41 million) occur due to NCDs, of which 17 million occur prematurely (before age 70). These deaths disproportionately affect people in low- and middle-income countries [1]. To combat increasing mortality from NCDs, projected to reach 52 million by 2030 [2], countries worldwide are working towards achieving Sustainable Development Goal 3.4, the aim of which is the reduction of premature mortality from NCDs by one-third by 2030 [1]. Several strategies implemented to reduce the burden of NCD risk factors showed limited effects. According to the World Health Organisation (WHO, 2024) risk factors for NCDs decreased by 4.67%; smoking reduced by 0.6%; hypertension reduced by 0.9%; while obesity and cholesterol increased 1.66% and 0.1%, respectively from 2015 to 2019, although there have been some advances in health care internationally, the changes in the risk factors or NCDs during the four years described were less than 2% of the global population [3].

Underlying social determinants such as socio-economic status, sociodemographic characteristics, geographical location, and rapid urbanisation contribute to the development of NCDs in rural-urban migration [4–6]. Within and between countries, individuals living in rural and low-resourced urban settings are exposed to various health determinants, including socio-economic status, sociodemographic characteristics, geographical locations, and rapid urbanisation [4–11]. Due to the differences in rural and urban living, social determinants affect these communities differently. The difference in rural and urban lifestyles leads to the development of risk factors for NCDs, such as physical inactivity, smoking, and unhealthy diets. Such diets are characterised by high consumption of salt, sugar and fat and a lack of consumption of fruits and vegetables. Consequently, unhealthy diets can lead to the development of obesity, hypertension, dyslipidaemia, and type 2 diabetes (biological or metabolic risk factors) [4, 10]. As a result, NCD risk factors vary depending on an individual’s geographical location and lifestyle choices, making populations in rural and urban areas susceptible to different health risks [12].

In an international context, physical inactivity was found to be significantly high in both rural and urban areas of Indonesia [13]. This was different from the Myanmar region, where physical inactivity was reported to be higher in rural areas [14]. Higher incidences of cardiovascular disease, diabetes, obesity, hypertension, hypercholesterolemia, and hypertriglyceridemia have been found in urban areas [13–15]. In South Africa, Peltzer et al. [7] found no rural-urban disparities in behavioural risk factors such as tobacco use, excessive alcohol consumption, physical inactivity and inadequate consumption of fruits and vegetables. On the contrary, Van Zyl et al. [16] found smoking and alcohol consumption to be more prevalent in rural areas of Free State Province compared with urban areas [16]. Cardiometabolic diseases are prevalent in both rural and urban areas of Free State Province, with hypertension and diabetes reported more frequently in rural areas [16]. Ajaero et al. [17] concluded that there are spatial disparities in NCD prevalence in rural and urban areas of South Africa, with urban areas showing higher prevalence (57%) than rural areas (43%), and this trend is consistent across all provinces and districts.

Physical inactivity, a modifiable risk factor for NCDs, is associated with decreased physical fitness [18–20], which is an indicator of functional performance and refers to an individual’s ability to carry out activities of daily living (ADL) safely, accurately, and independently without undue fatigue [19–22]. Objective functional performance, such as handgrip strength, walking speed, balance and sit-to-stand, are indicators of healthy biological ageing and are related to positive health outcomes [23]. Poor functional performance is associated with a loss of independence and a gradual decline in the ability to carry out ADL to the point of disability [24–27]. Furthermore, poor functional performance and the burden of NCDs can negatively impact one’s overall health-related quality of life (HRQoL) [11, 28, 29]. HRQoL is a multidimensional concept that focuses on the impact of NCDs on functional health and well-being as reported by the individual, or it is the individual’s perception of their life related to their culture and goals. It also involves physical health, psychological status, level of independence, and social and environmental relationships [29, 30].

Regarding functional performance and HRQoL disparities, John et al. [31] did not observe differences in ADL between rural and urban Canadian adults, which is similar to findings from the South African context [7]. Similarly, there were no variations in handgrip strength and subjective assessments of severe functional disability and QoL in a South African cohort [7]. On the contrary, rural Brazilian participants could carry out ADLs independently [32], while rural participants in Portugal exhibited higher functional performance and better HRQoL [9] compared with their counterparts in an urban setting. In other areas, such as China and India, higher HRQoL [33, 34] and functional performance [28] were reported in urban areas. Functional performance and HRQoL vary between rural and urban areas [33, 35]. Increasing rurality, being female, having low income and lower education levels, suffering from NCDs, and being of older age have all been associated with poor functional performance [31]. Conversely, good HRQoL has been associated with the ability to perform ADLs, absence of NCDs, being female, being unmarried, being unemployed, satisfaction with one’s living environment, and never smoking [11].

Although there is evidence of rural–urban disparities, inconsistencies exist regarding the distribution of NCD risk factors, functional performance and HRQoL within and between countries, making it challenging to compare results between rural and urban areas [12, 36, 37]. Limited research has been carried out on the disparities in risk factors for NCDs, functional performance and HRQoL between rural and urban areas worldwide, and existing studies cannot be generalised to all countries, including South Africa, due to varying socio-economic status and other social determinants [6, 8, 9, 31, 38, 39]. Furthermore, in the South African context, there has been a lack of comprehensive research on health disparities between rural and low-resourced urban areas [40]. As a result, there is a growing interest in understanding the health disparities associated with ageing among individuals from different socio-economic and environmental backgrounds, including rural and low-resourced urban residents [41, 42]. Therefore, understanding the distribution of NCD risk factors, functional performance and HRQoL in people living in rural and low-resourced urban settings offer valuable insight for crafting public health campaigns, including health promotion.

Therefore, this study aimed to examine disparities in risk factors for NCDs with objective measurement of physical inactivity, body composition, blood pressure, peripheral lipid and glucose profiles), functional performance (handgrip, single leg stand [SLS], sit-to-stand, timed-up-and-go [TUGS], predicted peak VO_2_ max), and HRQoL among adults from a rural and a low-resourced urban setting in South Africa.

## Methods

### Study design

A cross-sectional design was followed, analysing the baseline data from the overarching B-Healthy controlled trial (PACTR201609001771813; Date of registration 7 September 2016), which followed a pragmatic approach to determine the effect of an exercise intervention on risk factors for NCDs; medicine usage; functional performance; perceptions and knowledge of risk factors for NCDs and PA; as well as HRQoL among persons living in low-resourced urban and rural communities [43]. The B-Healthy study included the rural community of Vhembe District, Limpopo Province and Ikageng, a low-resourced urban setting in Potchefstroom, North West Province, South Africa for data collection.

### Participants

The convenience sampling method was used to recruit a total of 311 participants between the ages of 35 and 80 years (128 rural participants 183 low-resourced urban participants). Participants were recruited by distributing information flyers at surrounding churches, primary health clinics and public spaces that people frequently visited in the area of their designated primary health clinic. The rural and urban clinics provided the researcher space to collect data. Adults (35–80 years) with no physical impairments (ability to perform functional tests), relied on government-funded primary health clinics, had a stable clinical condition with at least one or more risk factors for NCDs ( overweight or obesity; hypertension; dyslipidaemia; prediabetes or diabetes; smoking; and/or sedentary lifestyle) were included in the study. All participants also completed a Physical Activity Readiness Questionnaire (PAR-Q) to ensure safe participation in the overarching B-Healthy exercise intervention study. Exclusion criteria was pregnant and/or lactating, absolute contraindications to exercise testing according to the American College of Sports Medicine (ACSM) guidelines [18], having psychological (unable to understand and respond to the PAR-Q) or physical limitations (identified from the PAR-Q screening), and access to private health care.

### Setting

Low-resourced urban participants were recruited in 2015 from two low-resourced communities in Ikageng, which is adjacent to Potchefstroom in Dr Kenneth Kaunda District, North West Province. The urban areas are typically located on the outskirts of towns or cities with limited access to built housing, running water, electricity, job opportunities and sanitation [44]. Rural participants were recruited in 2022 from two rural communities (Ha-Mutsha and Ha-Manavhela) in Vhembe District, Limpopo Province. Both of the rural locations are more than 30 km from the nearest town, Thohoyandou, and are almost 27 km apart. Both communities are governed under tribal law, and each has its own chief and civic committee that runs the community’s affairs. The population density of Ha-Mutsha and Ha-Manavhela is 992.17/ km^2^ and 1203.35/ km^2,^ respectively, compared to 5000/km^2^ of the low-resourced urban community [45]. The population of Ha-Mutsha is approximately 2800 black Africans, of which 97% are speaking Tshivenda as their first language. Ha-Manavhela, which is the second village, has a population of approximately 1800 (100% black African and 92% speaking Tshivenda as the first language) [45]. The common activities of these rural areas include small-scale farming (livestock and crops). Approximately 87,701 black Africans stay in this area, with 45% of the population being Setswana speaking, followed by 15% of Sesotho, and lastly by IsiXhosa (14%) [45].

### Demographic data

Sociodemographic information was collected with a questionnaire and included age (date of birth), gender, marital status, employment status, level of household income, level of education and family size.

### Risk factors for non-communicable diseases

#### Body composition measurements

All measurements were performed according to the International Society for the Advancement of Kinanthropometry (ISAK) guidelines, with participants wearing minimal clothing. The average of the three skinfold measurements was used [46]. Height and weight were accurately measured to the nearest 0.1 cm and 0.1 kg [46]. Body mass index (BMI) was calculated by dividing body weight in kilogrammes by height in metres squared (kg/m^2^) [46]. Participants were classified as normal weight (< 25 kg/m^2^), overweight (25–29.9 kg/m^2^) or obese (≥ 30 kg/m^2^) [18, 46]. Waist circumference (WC) as an NCD risk indicator was categorised as normal (female WC ≤ 88 cm, male WC ≤ 102 cm) or increased (female WC > 88 cm, male WC > 102 cm) [18, 46]. Skinfold measurements were taken on the right-hand side of the body. A three-site formula was used: chest, subscapular and triceps thickness for men, and triceps, suprailiac and abdominal thickness for women to determine body density, from which the percentage of body fat was calculated using Siri’s equation [18]:

$$\text{B}\text{o}\text{d}\text{y} \text{ f}\text{a}\text{t} \% = \left(\right\{[4.95/Db] - 4.50\} \times 100)$$

#### Assessment of biological risk factors

Blood pressure was measured on the left arm with an aneroid sphygmomanometer using the Riva-Rocci/Korotkoff method [47]. Participants were seated quietly (on a chair that supported their back and their feet flat on the floor) for at least 5 min before taking the reading on the arm supported at the level of the heart. The participants had refrained from smoking cigarettes and ingesting caffeine for at least 30 min prior to measurement. Two trials with a 5-minute resting interval were measured [48], and the average of the measurements was used in the analyses. Blood pressure was classified as normal (< 120/80 mmHg), elevated (120–129/<80 mmHg), and hypertension (≥ 130/>80 mmHg) [18]. Participants taking antihypertensive medication were also classified as hypertensive [18, 49].

Peripheral fasting lipid and glucose levels were measured using a minimally invasive finger prick. Measurements were taken in millimoles per litre (mmol/L) using glucose and cholesterol monitors (Accutrend, Roche Diagnostics, Germany) [50]. Raised blood lipids were classified as follows: total cholesterol (T-Chol) of ≥ 5.18 mmol/L, low-density lipoprotein cholesterol (LDL-Chol) of ≥ 3.37 mmol/L, and high-density lipoprotein cholesterol (HDL-Chol) of < 1.04 [18]. Blood glucose ≥ 5.5 mmol/L was classified as a risk factor [18]. Taking prescribed medication for diabetes and/or cholesterolemia resulted in classification as a risk factor [18, 49].

#### Habitual physical activity level assessment

PA was objectively measured using a combined accelerometer and heart rate device (ActiHeart®, CamNtech Ltd, Cambridge, UK) for seven consecutive days at 30-sec epochs [51]. After the procedural briefing, participants wore the ActiHeart® device on their chest to collect data on heart rate variability, activity energy expenditure, resting metabolic rate and total energy expenditure. The ActiHeart® device was calibrated prior to data collection using an 8-minute step test. Data were considered valid if a participant wore the device for a minimum of 4 days, of which one was a weekend day and at least 600 min per day [52]. The physical activity data was downloaded from the device and analysed using ActiHeart Software. The intensity of the PA was presented as time spent at different metabolic equivalents (METs), categorised based on the WHO guidelines on PA and sedentary behaviour (2022) as light (1–2.99 METs), moderate (3–5.9 METs), hard (6–8.9 METs) and very hard (≥ 9 METs). Aligned with the WHO guidelines (2020), the accumulated minutes of moderate-vigorous PA per week (MVPA) were used to classify the participants as inactive, insufficiently active (≤ 149 min of MVPA/week) or active (≥ 150 min of MVPA/week) [19].

### Functional performance assessment

To evaluate functional performance, cardiorespiratory fitness (predicted peak VO_2_ max), handgrip strength, 30-second sit-to-stand test, SLS test, and a TUGS were performed [51, 53].

#### Cardiorespiratory fitness assessment

The study estimated the cardiorespiratory fitness or peak oxygen uptake (peak VO_2_) using a submaximal graded 8-minute step test protocol with the ActiHeart® software version four (CamTech, UK) [51, 54]. The built-in ActiHeart® software extrapolates heart rate reading during the 8-minute step test to calculate the indirect peak oxygen uptake (peak VO_2_) in mL/min/kg. A metronome was used during the test to ensure participants adhered to the stepping rate, which increased from 15 to 33 steps per minute. After completing the test, the participants sat for 2 min while the recovery heart rate was recorded. The study followed contraindications to exercise testing and general indications to stop the test to ensure participant safety. Haemodynamic measurement during recovery was performed in a sitting position. The ActiHeart® device was removed only after completing the test [51, 54]. Participants were classified into quartiles according to the age-specific distributions of their relative VO_2_ peak attained during the test.

#### Strength and endurance assessment

Handgrip strength was used to measure the strength of the upper extremities with a hydraulic handgrip dynamometer (Takei Physical Fitness TKK 5401, Kogyo Co Ltd, Tokyo, Japan) [55]. Participants were seated on a chair with their upper arm alongside their trunk and their elbow flexed at a 90-degree angle [56, 57]. Participants were asked to exert a maximal force three times using the right and left hand, and the highest value of the best hand was used [35, 58]. Low resistance of the handgrip was defined as < 26 kg and < 18 kg for males and females, respectively [59].

Lower extremity endurance was measured using the 30-second sit-to-stand test. Participants were instructed to do as many sit-to-stands as possible in 30 s, with their arms folded across their chest, using a standard chair of 44 to 45 cm in height. The number of complete stands completed within the 30 s was recorded [60, 61]. Completing fewer than eight consecutive repetitions is associated with poor outcomes [21].

#### Static balance

The Single-leg-Stand (SLS) test was used to measure the static balance of the participants or the ability to stand on one leg for a prolonged period. Participants were instructed to stand upright on one leg (barefoot) while the other was placed against the inside knee of the standing leg, with hands on the hips. The time the participant can stand was taken to a maximum of 60 s [62].

#### Dynamic balance assessment

The TUGS test assessed motor coordination (dynamic balance and gait speed). The test consists of rising from an armless chair with a seat height of ~ 46 cm without using the arms, walking 9 m, returning to the chair and sitting again. The time to perform the test was recorded in seconds and converted to metres per second by dividing the total walked distance by time.

### Health-related quality of life assessment

A standardised Short Form (SF-8) questionnaire developed by Quality Metric Incorporated [63] with licence number QUO-02139-P8D4H4 was used to determine participants’ HRQoL. The SF-8 is a five- and six-point Likert scale questionnaire with eight items measuring health domains such as general health, bodily pain, physical functioning, physical role, vitality, social functioning, mental health and emotional. The SF-8 questionnaire was then summarised into two main components, the physical and mental components, which were calculated by weighting each SF-8 item using the norm-based criterion [64, 65]. The scoring for the SF-8 questionnaire ranged from 0 (the worst) to 100 (the best), with 50 and upward representing good HRQoL and 50 or below representing poor HRQoL [66, 67]. The questionnaire’s validity and reliability were previously established among Setswana-speaking adults from North West Province and presented Cronbach’s alpha of 0.87 and 0.87, respectively [68]. The 4-week SF-8 recall period was used for this study [69].

### Statistical analysis

The Statistical Package of Social Science (SPSS version 28) was used to analyse the data. Descriptive statistics were calculated for all variables. Visual inspection of histograms, quantile-quantile plots, and the Shapiro-Wilks test was performed to evaluate the normal distributions of the variables measured on a ratio scale. No imputations were made for missing data. Variables that conformed to a normal distribution were presented as mean and standard deviation (mean ± SD). Variables not normally distributed were reported as Median and 95% confidence intervals (95% CI). Categorical data were reported in frequencies and percentages. For normally distributed continuous data, an independent t-test was used to identify differences between the NCD risk factors, functional performance and HRQoL between rural and low-resourced urban communities’ participants. Mann-Whitney U-test was applied to determine differences between variables that were not normally distributed. Chi-square analyses determined the differences among categorical data between low-resourced urban and rural communities. The level of significance was set at *p* < 0.05.

## Results

### Participant characteristics

The participant characteristics are displayed in Table 1. A total of 311 (128 rural and 183 low-resourced urban) participants aged 35–80 years were included in the study, with a mean age of 52.84 ± 11.31 and 59.09 ± 10.69 years for rural and low-resourced urban, respectively. More females (84.2%) participated in the study than males (15.8%). Low-resourced urban residents were older (59.09 ± 10.69 vs. 52.84 ± 11.31 years; t(309) = 4.95, *p* = 0.001) and had a higher prevalence of tertiary education (34.3% vs. 5.0%; t(203.49) = -3.56, *p* = 0.001) than the low-resourced urban participants. The reported employment status was higher in rural communities (35.2%) than in low-resourced urbans (12.0%); however, 93% of the rural working class earned less than R50 000 per annum and only 7% earned between R50 000 and R250 000 per annum. Among employed low-resourced urban residents, 59.7% earned less than R50 000 and 40.3% earned above R50 000 annually. More than 60.1% of low-resourced urban families reported a high household composition (more than four people in a household), compared with 52.1% of rural families. The majority of low-resourced urban residents had reportedly been diagnosed with hypertension; in contrast, rural residents with diagnosed hypertension were a minority (*p* = 0.001).

**Table 1:** Descriptive characteristics of rural and low-resourced urban participants

	Rural		Low-resourced urban		*p*-value	
	*n*	M ± SD	*n*	M ± SD		
**Age (years) (M ± SD)**	128	52.84 ± 11.31	183	59.09 ± 10.69	< 0.001^**^	
**Gender, n (%)**	**n**	**%**	**n**	**%**	**p-value**	
Male	31	24.2	18	9.8	< 0.001^**^	
Female	97	75.8	165	90.2		
**Highest level of education, n (%)**						
No schooling	41	32	55	30.4	< 0.001^**^	
High school	43	33.6	117	64.6		
Tertiary	44	34.4	9	5		
**Employment status, n (%)**						
Employed	45	35.2	23	12.6	< 0.001^**^	
Unemployed	35	27.3	85	46.4		
Unable to work or retired	48	37.5	75	41		
**Marital status, n (%)**						
Married	77	60.2	77	42.8	0.008^*^	
Single	26	20.3	48	26.7		
Widowed	15	11.7	43	23.9		
Divorced	10	7.8	12	6.7		
**Number of household members, n (%)**						
1–3	57	47.9	73	39.9	< 0.001^**^	
4–6	62	52.1	90	49.2		
> 6	0	0	20	10.9		
**Household income per annum, n (%)**						
< R50 000	119	93	108	59.7	< 0.001^**^	
R50 000–R250 000	9	7	58	32		
> R250 000	0	0	15	8.3		
**Reported diagnosed medical condition, n (%)**						
Not diagnosed	73	57.5	55	30.1	< 0.001^**^	
Hypertension	40	31.5	82	44.8		
Diabetes	6	4.7	6	3.3		
Hypertension and diabetes	8	6.3	40	21.9		

M = Mean; SD = Standard deviation. *Significant difference *p* ≤ 0.05; **Significant difference *p* ≤ 0.001.

The differences between low-resourced urban and rural participants are displayed in Table 2. Low-resourced urban residents exhibited significantly higher average values for body fat percentage (t(293.87) = 21.80, *p* = 0.001), T-Chol (t(288) = 3.64, *p* = 0.001), diastolic blood pressure (DBP) (t(296.66) = 2.35, *p* = 0.02), HDL-Chol (t(285.97) = 2.01, *p* = 0.05) and LDL-Chol (t(265) = 2.65, *p* = 0.01), and lower MVPA per week (t(103.69) = -3.01, *p* = 0.003), than the rural participants. Rural participants were significantly taller (1.61 ± 0.08 m vs. 1.56 ± 0.08 m; t(307) = -5.52, *p* = 0.001) with a lower body fat percentage (12.23% ± 4.67% vs. 27.69% ± 7.65%) compared with low-resourced residents. Although no significant difference in BMI was found between the rural and low-resourced urban communities, the average BMI for both rural (30.58 ± 7.38 kg/m^2^) and low-resourced urban (31.50 ± 7.89 kg/m^2^; t(307) = 1.03, *p* = 0.304) participants was higher than the healthy norms prescribed by the American College of Sports Medicine (ACSM) [28]. The average SBP for both rural (130.75 ± 20.11 mmHg) and low-resourced urban (135.40 ± 21.53 mmHg; t(309) = 1.93, *p* = 0.06) was classified as hypertensive and was borderline statistically significantly higher in the low-resourced urban group [28]. Although not exhibiting statistically significant differences between the two groups, mean glucose levels for both populations fell into the pre-diabetes category. Mean lipid profiles for both communities remained within the normal range but significantly differed between the rural and low-resourced urban groups [18]. Objectively measuring PA was used to determine the amount of PA and classify communities as active or inactive. Neither community met the minimum recommendation for PA of at least 150 min/week of MVPA. However, rural residents demonstrated significantly higher average moderate to vigorous PA.

**Table 2:** Differences in risk factors for non-communicable diseases between rural and low-resourced urban communities

NCD risk factors	Rural		Low-resourced urban		*p*-value
	*n*	M ± SD	*n*	M ± SD	
BMI (kg/m^2^)	128	30.58 ± 7.38	181	31.50 ± 7.89	0.30
Waist (cm)	128	92.32 ± 15.85	176	92.19 ± 13.68	0.94
Body fat (%)	128	12.23 ± 4.67	176	27.69 ± 7.65	< 0.001^**^
SBP (mmHg)	128	130.75 ± 20.11	183	135.40 ± 21.53	0.06
DBP (mmHg)	128	79.89 ± 11.93	183	83.36 ± 13.95	0.02^*^
Glucose (mmol/L)	127	6.06 ± 2.76	163	5.56 ± 3.0	0.15
T-Chol (mmol/L)	127	3.80 ± 1.03	163	4.23 ± 0.97	< 0.001^**^
HDL-Chol (mmol/L)	127	1.33 ± 0.36	161	1.43 ± 0.45	0.05^*^
LDL-Chol (mmol/L)	122	1.72 ± 0.86	145	2.00 ± 0.87	0.01^*^
Triglyceride (mmol/L)	126	1.70 ± 1.05	162	1.87 ± 0.98	0.16
Chol-HDL-ratio (mmol/L)	127	3.0 ± 1.01	153	3.10 ± 0.91	0.39
**Physical activity outcome**	**n**	**M ± SD (95% CI)**	**n**	**M ± SD (95% CI)**	**p-value**
MVPA (min/week)	65	62.92 ± 60.43(47.95–77.90)	147	37.19 ± 49.55 (29.1245.27)	0.003^*^

BMI = Body mass index; DBP = Diastolic blood pressure; HDL-Chol = High-density lipoprotein cholesterol; LDL-Chol = Low-density lipoprotein cholesterol; M = Mean; MVPA = Moderate-vigorous physical activity; NCD = Non-communicable disease; SBP = Systolic blood pressure; SD = Standard deviation; T-Chol = Total cholesterol.

*Significant difference *p* ≤ 0.05; **Significant difference *p* ≤ 0.001.

**Fig. 1:**
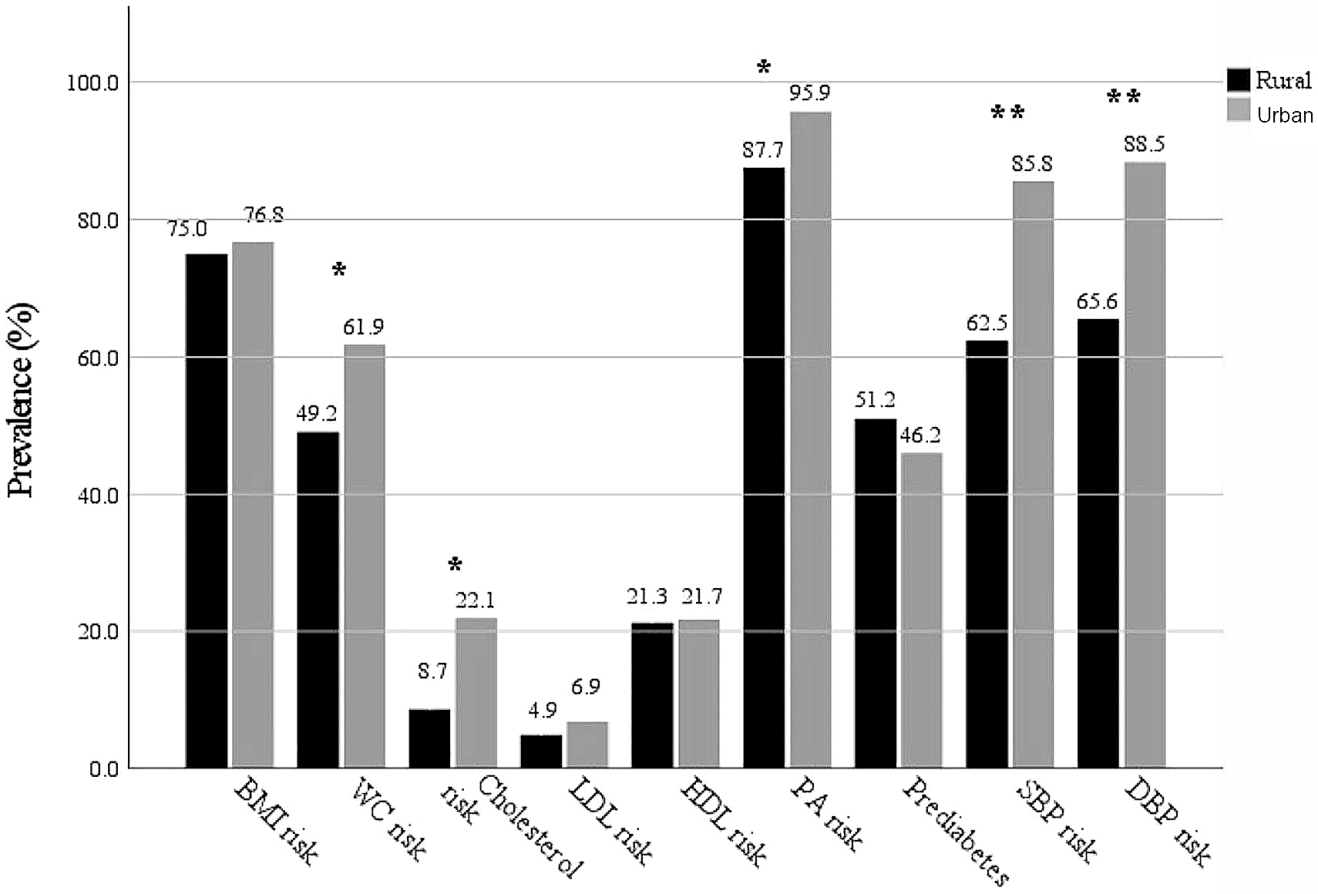
The prevalence of participants with non-communicable disease risk factors according to rural or low-resourced urban setting. DBP = Diastolic blood pressure; HDL-Chol = High-density lipoprotein cholesterol; LDL-Chol = Low-density lipoprotein cholesterol; PA = Physical inactivity; SBP = Systolic blood pressure; T-Chol = total cholesterol; WC = Waist circumference. *Significant difference *p* ≤ 0.05; **Significant difference *p* ≤ 0.001

The prevalence of the top four NCD risk factors in rural areas ranked from highest to lowest was (Fig. 1): PA inactivity (87.7%), increased BMI (75.0%), DBP ≥ 80 mmHg (65.6%) and SBP ≥ 130 mmHg (62.5%). In the low-resourced urban area, the top four ranked NCD risk factors from highest to lowest were as follows: PA inactivity (95.9%), DBP (88.5%), SBP (85.8%) and increased BMI (76.8%). In the prevalence of NCDs, participants in low-resourced urban areas had a statistically significant higher prevalence of larger WC (*p* = 0.027), elevated SBP (*p* = 0.001), elevated DBP (*p* = 0.001), elevated T-Chol (*p* = 0.002) and PA inactivity (*p* = 0.026) than participants from the rural setting.

In Table 3, the functional performance of participants from rural and low-resourced urban areas is presented and shows statistically significant differences between the two areas in most functional performance indicators, including the SLS test (right: t(282) = -10.68, *p* < 0.001; left: t(281) = -11.77, *p* = 0.001) and predicted peak VO_2_ max (t(262) = -6.52, *p* = 0.001). In nearly all functional performance indicators, rural residents outperformed their low-resourced urban counterparts, except for the 30-second sit-to-stand test, scoring slightly lower on average.

**Table 3:** Functional performance by rural–low-resourced urban residence

Physical function parameters	Rural		Low-resourced urban		*p*-value
	*n*	M ± SD	*n*	M ± SD	
Right HG (kg)	128	26.17 ± 8.17	166	25.54 ± 6.93	0.48
Left HG (kg)	126	25.37 ± 8.17	164	25.15 ± 6.27	0.80
SLST-Right (sec)	125	44.96 ± 18.47	159	20.87 ± 19.18	< 0.001^**^
SLST-Left (sec)	125	46.76 ± 17.95	158	20.70 ± 18.92	< 0.001^**^
30-second sit-to-stand test (n)	124	11.84 ± 2.53	162	12.43 ± 4.58	0.17
Timed-up-and-go speed (m/sec)	123	1.11 ± 0.94	164	1.03 ± 0.25	0.40
Predicted peak VO_2_ max (mL/min/kg)	121	23.99 ± 9.89	143	16.95 ± 7.64	< 0.001^**^

HG = Handgrip strength; SLST = Single leg stand test.

*Significant difference *p* ≤ 0.05; **Significant difference *p* ≤ 0.001.

Table 4 shows the distribution of HRQoL subdimension and main component summary. Rural residents exhibited statistically higher average values in all eight HRQoL subdimensions and in the general summaries of the physical and mental components. Rural participants reported better HRQoL than low-resourced urban residents, who scored poorly in all majors except vitality.

**Table 4:** Differences between health-related quality of life of rural–low-resourced and urban residence

SF-8 HRQoL	Rural		Low-resourced urban		*p*-value
	*n*	M ± SD	*n*	M ± SD	
**Physical component summary**	128	51.06 ± 8.14	182	45.62 ± 11.13	< 0.001^**^
Physical functioning	128	51.49 ± 6.41	182	46.24 ± 10	< 0.001^**^
Role physical	128	51.92 ± 6.12	182	46.90 ± 9.91	< 0.001^**^
Body pain	128	48.69 ± 12.10	182	45.32 ± 12.3	0.02^*^
General health	128	49.14 ± 10.67	182	43.16 ± 10.19	< 0.001^**^
**Mental component summary**	128	54.75 ± 8.24	182	48.91 ± 12.27	< 0.001^**^
Mental health	128	52.95 ± 8.51	182	47.37 ± 12.09	< 0.001^**^
Vitality	128	55,39 ± 8.84	182	51.02 ± 10.16	< 0.001^**^
Social functioning	128	53.63 ± 5.27	182	48.53 ± 9.47	< 0.001^**^
Role emotional	128	50.88 ± 4.85	182	47.34 ± 8.42	< 0.001^**^

HRQoL = Health-related quality of life; SF-8 = Short Form HRQoL questionnaire.

The scoring of SF-8 ranged from 0 (the worst) to 100 (the best).

^*^Significant difference *p* ≤ 0.05; ^**^Significant difference *p* ≤ 0.001.

## Discussion

This study aimed to determine the differences in NCD risk factors, functional performance and HRQoL of adults (35–80 years) in rural and low-resourced urban communities in South Africa. Overall, there were significant differences between the point prevalence of NCD risk factors such as high blood pressure, physical inactivity, high total cholesterol, increased WC, average functional performance values for single leg stand and CRF and HRQoL, including the mental and physical components. Physical inactivity was the leading risk factor among participants in rural and low-resourced urban settings, followed by hypertension. The average BMI of participants from both settings indicated obesity, with the mean WC value indicating excess fat deposition, primarily in the upper extremities.

Although physical inactivity was the most prevalent risk factor in both communities, there were significant differences in its prevalence between the rural and low-resourced urban communities. The reason for the differences may be due to differences in living conditions. It is already established that residents in rural communities of low-income status are more likely to engage in full-time, labour-intensive farming as a primary source of livelihood and perform household chores manually, compared with people from urban areas of higher income, who often rely on motor vehicles for transport and household appliances such as washing machines to complete domestic chores, which decreases their overall level of PA [70, 71]. Low-resourced urban participants were on average older, with a higher household income and a lower educational level than rural residents. These results aligned with previous research findings that indicated [14, 35], individuals with a higher income and lower educational levels exhibit a higher level of physical inactivity than their poorer and more educated counterparts [31, 36].

The findings of this study were consistent with studies in South Africa and internationally that indicated that rural residents tend to be more physically active than urban dwellers [16, 70, 72]. However, in our study a high prevalence of physical inactivity was found in both rural and low-resourced urban participants. The findings of Peltzer et al. [7] in a South African national sample and Htet et al. [14] in Yagoon, Myanmar differed from the current study’s findings. Neither found any significant differences in physical inactivity between rural and urban communities, although a possible reason for this may be explained by their use of self-reported methods to determine PA levels. Our study objectively measured PA, which has been found to have greater construct validity than self-report measures, particularly in adults [73]. Additionally, the trend of a global increase in physical inactivity might also contribute to this difference in findings. Additionally, it is important to note that the urban area data in the study of Peltzer and colleagues [7] was obtained from a well-resourced urban area. In contrast, the present study utilised data from a low-resourced urban area [7, 74].

Elevated blood pressure or hypertension was the second most prevalent risk factor in both communities, with low-resourced urban residents having a significantly higher prevalence than rural residents. The plausible explanation for the higher prevalence could be the increased rate of physical inactivity in low-resourced urban communities, older age, the higher waist circumferences as an indicator of central obesity, and higher household income [14]. Although we did not collect dietary intake data, urban populations tend to consume higher volumes of calorie-dense food than rural communities, which contributes to the obesity prevalence and subsequent increases in blood pressure. These findings differed from those of Van Zyl et al. [16] in Free State Province, South Africa and Rosjidi et al. [13] in Indonesia, who both reported a significantly higher prevalence of hypertension in rural areas compared with urban areas. Contrary to the latter results, Peltzer et al. [7] reported no significant differences between rural and urban communities in hypertension in a national sample from South Africa. In an international context, our findings were consistent with those of Htet et al. [14], who reported a high prevalence of hypertension among urban residents in the Yangon region of Myanmar. The differences in hypertension prevalence observed in the present investigation compared with those reported by Van Zyl et al. [16] and Rosjidi et al. [13] could be attributable to the distinction between urban and low-resourced urban settings. Furthermore, our study used the new blood pressure classification of ≥ 130/>80 mmHg, rather than ≥ 140/>90 mmHg [18, 49], which Van Zyl and Rosjidi used.

In this study, rural and low-resourced urban residents exhibited average BMI values that categorise them as obese [18]. Additionally, low-resourced urban residents showed a significantly higher prevalence of elevated WC and hypercholesterolemia, and higher average body fat percentage values. These differences could account for the increased prevalence of high blood pressure in the low-resourced urban residents compared with the rural residents, who, conversely, had a significantly lower prevalence of elevated WC and hypercholesterolemia, and lower average values for body fat percentage. Physical inactivity coupled with excess body fat especially concentrated around the abdominal region, is associated with an increased risk of metabolic risk factors such as hypertension and dyslipidaemia [12, 18]. Different to this study, Van Zyl et al. [16] found no significant differences in the prevalence of elevated WC in rural and urban areas of Free State Province in South Africa. Furthermore, hypercholesterolemia was significantly higher in rural areas than in urban areas [16]. A potential explanation for Peltzer’s different results may be their focus on the elderly population in a high-resource urban area rather than in a low-resourced urban.

Physical fitness and PA levels are two valid indicators strongly associated with functional performance and they predict autonomy, morbidity and mortality in adults [35]. This study demonstrated higher functional performance in rural residents than in their low-resourced urban counterparts. Despite not meeting the minimum recommendation of more than 150 min spent in MVPA, rural participants exhibited significantly higher average values for MVPA time, SLS test score and peak VO_2_ max values than the low-resourced urban participants. These differences may be linked to differences in age and ADL. These results are in agreement with Furtado et al. [9], who attributed these significant differences in rural functional performance to routine activities requiring muscle strength and endurance in the upper and lower extremities, such as caring for animals and managing small farms. Contrary to our findings, Alex et al. [28] in India found no rural–urban differences in the functional status of middle-aged to older adults in rural and urban Indian communities. A possible explanation for the differences between our result and that of Alex et al. [28] may be the participants’ age and setting; their focus was on an elderly population aged 60 to 75 years living in high-resourced urban areas. Our study focused on a wider age group, 35–80 years old, residing in a low-resourced area.

Rural residents exhibited significantly higher HRQoL compared with their low-resourced urban counterparts. Rural residents scored above the minimum cut-off point of 50% for good HRQoL for all measures except bodily pain and general health, which were slightly below 50%. Low-resourced urban residents reported poor HRQoL (< 50 total score) for all measures except vitality. Lower scores in rural dwellers for bodily pain and general health may be associated with the physical demands of manual labour, potentially resulting in increased musculoskeletal discomfort and the lack of adequate healthcare access, which could result in untreated pain. In low-resourced urban settings, lower HRQoL scores may be explained by the older age of participants. Several studies have illustrated a positive relationship between functional performance and HRQoL [9, 28]. Therefore, improved rural functional performance can contribute to the significantly higher average values for all HRQoL subdimensions and main component summary scores. These results corroborate the findings of Peltzer and Pengpid et al. [75] and Prasad et al. [35] that PA is a determinant of functional performance, which decreases with increasing age, leading to increased functional disability. This result is corroborated with those of Furtado et al. [9] in Portugal, who reported that rural residents had higher scores for HRQoL and functional fitness than urban adults, with significant differences in all subdimensions of HRQoL except for general health, mental health and changes in health status. Contrary to our findings, Peltzer et al. [16] did not find rural-urban differences in QoL and severe functional disability in rural and urban South Africans [16]. In China [33] and India [34], HRQoL was higher in urban than rural areas. These differing results from our study may be due to differences in settings, such as urban versus low-resourced urban.

We found that low-resourced urban residents were generally older, had higher unemployment and a larger household size, and exhibited a higher prevalence of NCD risk factors, which could potentially explain their lower functional performance and HRQoL compared with rural residents. Rural dwellers, slightly younger with higher employment but lower income, had a lower risk factor prevalence and engaged in more PA, possibly explaining their better health outcomes. However, both communities had a widespread prevalence of risk factors, such as high levels of obesity according to their BMI, and did not meet the recommended minimum level of PA.

In this study, we focused on examining disparities in health-related aspects between two regions to understand the health disparities in South Africa clearly. Further research is required to explore the connection between NCD risk factors, functional performance and HRQoL in rural and low-resourced urban communities.

The strength of this study lies in the objective measurement of PA levels in low socio-economic settings for both rural and urban communities. To our knowledge this is the first study to report on objectively determined PA in persons with at least one risk factor for NCDs, as well as the differences observed between rural and urban communities with limited resources. This study’s findings must be interpreted against the limitations that were present. A convenience sampling method was employed due to the pragmatic approach of the overarching B-Healthy study, which limits the generalisability of the findings to other communities. Although there was a time lapse between the data set from the low-resourced urban and rural communities, socio-economic aspects within the communities did not change significantly during this period, therefore, the results are still valuable, and offer important insights into the disparities between the two settings. The limited number of male participants willing to participate in the study prevented gender comparisons within the study.

## Conclusion

The study concludes that differences exist in NCD risk factors, functional performance and HRQoL between rural and low-resourced urban communities. The low-resourced urban community presented a higher prevalence of elevated blood pressure, physical inactivity, T-Chol, and WC than rural residents. The average values for rural residents’ functional performance, single-leg stand and CRF, were significantly higher compared to the low-resourced urban residents. Furthermore, rural participants experienced a better overall HRQoL compared to low-resourced urban areas. Although there were significant differences, neither community met the minimum recommendation for PA, and low-resourced urban residents reported a poor score in all subdimensions of HRQoL except vitality. Future research should investigate the associations between the risk factors of NCD, functional performance, and HRQoL in order to develop intervention programs that are appropriate and tailored to the specific community.

## Data Availability

Relevant data from this study will be made available upon study completion and researchers request from thecorresponding author.

## Abbreviations

ACSM: American College of Sports Medicine
ADL: Activities of daily living
BMI: Body mass index
DBP: Diastolic blood pressure
HDL-Chol: High-density lipoprotein
HG: Handgrip
HRQoL: Health-related quality of life
LDL-Chol: Low-density lipoprotein cholesterol
ISAK: International Society for the Advancement of Kinanthropometry
METs: Metabolic equivalents
MVPA: moderate-vigorous physical activity
NCD: Non-communicable disease
NWU: North-West University
PA: Physical activity
PAR-Q: Physical Activity Readiness Questionnaire
QoL: Quality of life
SBP: Systolic blood pressure
SF-8: Short form questionnaire
SLS: Single leg stand
T-Chol: Total cholesterol
TUGS: Timed-up-and-go speed
WC: Waist circumference
WHF: World Heart Federation
WHO: World Health Organization
